# Freely Generated Word Responses Analyzed With Artificial Intelligence Predict Self-Reported Symptoms of Depression, Anxiety, and Worry

**DOI:** 10.3389/fpsyg.2021.602581

**Published:** 2021-06-04

**Authors:** Katarina Kjell, Per Johnsson, Sverker Sikström

**Affiliations:** Department of Psychology, Lund University, Lund, Sweden

**Keywords:** diagnostic criteria, major depressive disorder, generalized anxiety disorder, measurement, artificial intelligence, natural language processing, machine learning, diagnostic assessment

## Abstract

**Background:**

Question-based computational language assessments (QCLA) of mental health, based on self-reported and freely generated word responses and analyzed with artificial intelligence, is a potential complement to rating scales for identifying mental health issues. This study aimed to examine to what extent this method captures items related to the primary and secondary symptoms associated with Major Depressive Disorder (MDD) and Generalized Anxiety Disorder (GAD) described in the Diagnostic and Statistical Manual of Mental Disorders (DSM-5). We investigated whether the word responses that participants generated contained information of all, or some, of the criteria that define MDD and GAD using symptom-based rating scales that are commonly used in clinical research and practices.

**Method:**

Participants (*N* = 411) described their mental health with freely generated words and rating scales relating to depression and worry/anxiety. Word responses were quantified and analyzed using natural language processing and machine learning.

**Results:**

The QCLA correlated significantly with the individual items connected to the DSM-5 diagnostic criteria of MDD (PHQ-9; Pearson’s *r* = 0.30–0.60, *p* < 0.001) and GAD (GAD-7; Pearson’s *r* = 0.41–0.52, *p* < 0.001; PSWQ-8; Spearman’s *r* = 0.52–0.63, *p* < 0.001) for respective rating scales. Items measuring primary criteria (cognitive and emotional aspects) yielded higher predictability than secondary criteria (behavioral aspects).

**Conclusion:**

Together these results suggest that QCLA may be able to complement rating scales in measuring mental health in clinical settings. The approach carries the potential to personalize assessments and contributes to the ongoing discussion regarding the diagnostic heterogeneity of depression.

## Introduction

Closed-ended rating scales are commonly used in clinical practice and research to assess the type and severity of mental health issues [e.g., the Patient Health Questionnaire-9 (PHQ-9); Kroenke et al., 2001, and the Generalised Anxiety Disorder Scale-7 (GAD-7); Spitzer et al., 2006]. These rating scales require the respondent to rate their agreement with predefined items designed to target the construct/disorder being measured.

Question-based computational language assessment (QCLA) is an alternative method to rating scales (Kjell et al., 2019). This method has an open-ended word-response format that allows the respondent to freely elaborate on their state of mind using descriptive words or texts that are analyzed using natural language processing and machine learning. Previous research shows that QCLA *measures*, *describes*, and *differentiates* well between psychological constructs (Kjell et al., 2019) when compared with the total score of rating scales specifically designed to capture the Diagnostic and Statistical Manual of Mental Disorders (DSM-5) criteria (American Psychological Association [APA], 2013). This study aimed to further investigate the QCLA method by examining to what extent it captures individual items related to the primary and secondary symptoms associated with mental health aspects described in the DSM using the PHQ-9 (Kroenke et al., 2001) and the GAD-7 (Spitzer et al., 2006). These rating scales are designed to target the DSM criteria for Major Depressive Disorder (MDD) and Generalized Anxiety Disorder (GAD; American Psychological Association [APA], 2013).

### Computational Language Assessment

Computational language assessments have been used to predict and monitor depression on a population level using *naturally occurring text* on social media (e.g., Mowery et al., 2016). Posts on social media have also been used to predict further episodes of depression several months before onset using Twitter (De Choudhury et al., 2013; Reece et al., 2017) and Facebook (e.g., Eichstaedt et al., 2018). Eichstaedt et al. (2018) predicted depression as recorded in participants’ medical records using language from Facebook posts. They further predicted episodes of depression 3 months before they were documented in the medical records, suggesting that prediction models based on social media might be a useful complement in diagnostic screening procedures (Eichstaedt et al., 2018). However, less research has been done when it comes to QCLA where participants are asked about aspects of their mental health.

### Question-Based Computational Language Assessment

Kjell et al. (2019) constructed QCLAs with the aim of measuring and describing mental health, including depression, worry, harmony in life, and satisfaction with life, and evidence suggests that this method quantifies constructs with similar or greater validity compared with traditional rating scales. For example, in two studies participants were asked to describe facial expressions from a validated database, and it was found that QCLAs accurately categorized significantly more facial expressions compared with rating scales. It was further demonstrated that QCLAs of subjective experience predict rating scales’ total score with correlations of *r* = 0.58 for the GAD-7, *r* = 0.59 for the PHQ-9, *r* = 0.72 for the Harmony in life scale, and *r* = 0.63 for the Satisfaction with life scale (*p* < 0.001 for all *r*-values; *N* = 477). In another study, it was demonstrated that the QCLA of harmony in life was significantly correlated with cooperative behavior (*r* = 0.18 for all participants and *r* = 0.35 for participants categorized as prosocial); whereas the corresponding rating scale of harmony in life (Kjell et al., 2021a) did not demonstrate a significant correlation (Kjell et al., 2021a).

#### The QCLA Method

The word or text responses generated from questions on QCLAs are suitable for statistical analyses based on the creation of high-dimensional word embeddings from a large language corpus. The QCLA method quantifies words, or texts, as a vector, i.e., the word embedding of word responses, and uses this word embedding to construct three types of measures: *semantic similarity scales*, *language-trained scales*, and *language-predicted valence scales* (Kjell et al., 2019).

##### Semantic similarity scale

The semantic similarity scale has the advantage of being able to measure a construct based on an empirically generated semantic definition of a construct. This is achieved by creating a word norm (a list of empirically derived words) that participants have generated to describe the construct being measured. The semantic similarity scale is measured by the semantic similarity (closeness) between the participants’ word responses and the targeted word norm. This procedure is carried out without any involvement of a rating scale.

Word norms may describe the two endpoints of a psychological construct e.g., “being depressed” or “not at all depressed” (Kjell et al., 2020). A unipolar semantic similarity scale is the semantic similarity between word responses and *one* word norm (e.g., depression responses and the being depressed word norm). A bipolar semantic similarity scale is the semantic similarity between the text generated from the to-be-measured questions and the similarity scores between the subtraction of two word norms (e.g., depression responses to the being depressed word norm minus the depression responses to the not at all being depressed word norm). Unipolar and bipolar semantic similarity scores for depression and worry have been found to correlate well with the total scores of rating scales, where bipolar scores correlate stronger than unipolar scores (Kjell et al., 2020).

##### Language-trained scale

The language-trained scale can be used to measure a construct using word or text data by linking it to well-established rating scales or other quantifiable markers related to the concept. For example, the word embedding generated by a word-response question about depression can be used to predict the rating scale score of the PHQ-9. This prediction can be done, for example, by using multiple linear regression or other machine learning methods, and the validity can be evaluated with cross-validation methods. The measure of accuracy of the cross-validated predictions is calculated by the correlation between the predicted and actual scores.

##### Language-predicted valence scale

It is possible to take a prediction model trained on one dataset and apply it to another dataset. Valence is an important dimension on which emotions can be described and identified (e.g., Bradley and Lang, 1999). Kjell et al. (2019) trained a valence model using the Affective Norms for English Words, where participants have rated the emotional valence ranging from unpleasant to pleasant of more than 1,000 words (ANEW; Bradley and Lang, 1999). The model was then used to predict the valence scores of word responses, where the predicted valence scores were found to be strongly correlated with rating scale scores.

#### Semantic Similarity Scales Versus Language-Trained Scales

A language-trained scale can be trained to estimate a rating scale. However, this scale is typically constructed with different items, where some items can be predicted with higher accuracy than other items. In contrast, semantic similarity scales rely on the agreement between how respondents answering the word-response questions and how respondents creating the word norm understand the construct being measured. Thus, there is a fundamental difference between these that we investigate further.

### QCLA in the Clinical Setting

A response format where a person describes their mental health with their own words has many potential advantages in clinical practice. For example, it allows for patient-centered care because it focuses on the patient’s unique set of symptoms. Further, QCLA may add knowledge for a more patient-centered approach to routine outcome measures used in everyday clinical practice to monitor symptom severity and treatment effectiveness (e.g., see de Beurs et al., 2011; Washington and Lipstein, 2011). QCLA may also identify co-occurring symptoms that otherwise would have been undetected or may increase awareness of domains that are important to patients but that are not targeted, or captured, in rating scales.

#### Measuring Psychiatric Disorders Versus Subjective Well-Being

The open-ended nature of the QCLA method taps into an interesting difference between the measurement of psychiatric disorders versus subjective well-being. Assessments of psychiatric disorders (e.g., DSM-5, ICD-10) are strictly criteria driven, whereas measures of subjective well-being aim to be criteria free (e.g., Kesebir and Diener, 2008; Kjell and Diener, 2020). Corresponding to this, the open-ended word response format of word-response questions enables respondents to express themselves more freely than when responding to closed-ended rating scales. In contrast, diagnosing individuals with psychiatric disorders involves assessing whether individuals fulfill a specific set of diagnostic criteria stated in manuals such as the DSM-5 (American Psychological Association [APA], 2013). Hence, these approaches differ in whether it is the patients/clients or the professionals who specify the evaluation criteria. In subjective well-being measures, it is the respondent who is assumed to be best suited to judge their level of well-being (Kesebir and Diener, 2008); whereas for psychiatric disorders it is a trained researchers or mental health care professionals who define if someone meets the specified diagnostic criteria (American Psychological Association [APA], 2013). This difference makes it important to understand the word-responses’ relationship to individual rating scales’ items of criteria-based mental health disorders.

### DSM Criteria

The DSM-5 categorizes the criteria into *primary* and *secondary* for both MDD and GAD. For an individual to be diagnosed with MDD, they have to meet one of the primary criteria and five in total (including the primary and secondary criteria). To be diagnosed with GAD an individual has to meet all of the primary criteria and at least three of the secondary criteria. The rating scales used in this study are explicitly designed to capture the symptoms and criteria outlined in the DSM (e.g., for depression this includes disabilities in areas such as sleep, concentration, and movement; Kroenke et al., 2001). On the other hand, the QCLA questions only focus on assessing the respondent’s own understanding of depression (e.g., *Over the last 2 weeks, have you been depressed or not?*) and does not explicitly probe about specific symptoms that respondents not explicitly report following the question about being depressed. Thus, indirect symptoms such as changes in sleep, concentration, or movements can, but do not necessarily need to be reported. Hence, it is important to understand to what extent the broad question currently used in QCLA also captures specific symptoms and to what extent it might be necessary to also ask respondents specific symptom-related word-response questions.

#### Major Depressive Disorder

The two *primary* DSM-5 criteria for MDD focus on the subjective experiences of depression (i.e., depressed mood and loss of interest), and the *secondary* criteria focus on related symptoms such as psychomotor agitation or retardation, diminished ability to concentrate, and weight loss/gain. Hence, the primary symptoms are arguably closer to how individuals primarily think about depression, whereas individuals might not directly associate the secondary criteria as strongly with being depressed.

Reviewing the PHQ-9 shows that the nine items capture each of the DSM-5 diagnostic criteria well (see Table 1). In contrast, the QCLA for depression captures individuals’ subjective experiences and their own understanding of depression, which potentially might be more related to the primary rather than the secondary criteria. That is, instructions for the QCLA for depression (and worry) are broad and generally stated: “*Write descriptive words relating to those aspects that are most important and meaningful to you*” (see the section “Materials and Methods” for full details; Kjell et al., 2019).

**TABLE 1:** DSM-5 diagnostic criteria for Major Depressive Disorder.

**DSM-5 criteria**	**Primary**	**PHQ-9 items**
Five or more symptoms including depressed mood and/or loss of interest or pleasure, during a 2-week period.		
Depressed mood (e.g., feels sad, empty, hopeless, tearful).	Y	*Item 2*. Feeling down, depressed, or hopeless.
Markedly diminished interest or pleasure.	Y	*Item 1*. Little interest or pleasure in doing things.
Weight loss when not dieting or weight gain.	N	*Item 5*. Poor appetite or overeating.
Insomnia or hypersomnia.	N	*Item 3*. Trouble falling or staying asleep, or sleeping too much.
Psychomotor agitation or retardation (observable by others).	N	*Item 8*. Moving or speaking so slowly that other people could have noticed? Or the opposite—being so fidgety or restless that you have been moving around a lot more than usual.
Fatigue or loss of energy.	N	*Item 4*. Feeling tired or having little energy.
“Feeling worthless or excessive, delusional or inappropriate guilt.	N	*Item 6*. Feeling bad about yourself—or that you are a failure or have let yourself or your family down.
Fogginess, being unfocused, or indecisive.	N	*Item 7*. Trouble concentrating on things, such as reading the newspaper or watching television.
Thought about harming yourself or suicide.	N	*Item 9.* Thoughts that you would be better off dead or of hurting yourself in some way.

*PHQ-9: Patient Health Questionnaire 9 (Kroenke et al., 2001).*

*DSM-5: Diagnostic and Statistical Manual of Mental Disorders.*

*Primary symptoms are marked with Y.*

#### Generalised Anxiety Disorder

The two *primary* DSM-5 criteria for GAD focus on the experience of excessive worry and having difficulties in controlling one’s worrying, whereas the *secondary* criteria mainly focus on related symptoms such as muscle tension, irritability, and fatigue. Also, as for MDD, it can be argued that individuals may focus on the primary, rather than the secondary criteria, when answering the broad word-response questions.

The GAD-7 is developed to capture the DSM-5 criteria for GAD, although the scale does not include items for all symptoms. In addition, it also includes items that are not part of the DSM-5 criteria (for details, see Table 2). In contrast, the Penn State Worry Questionnaire-Abbreviated, (PSWQ-8; Hopko et al., 2003) focuses more on worry than the GAD-7. All eight items in the PSWQ-8 include the construct worry (i.e., *worries*, *worry*, *worrying*, or *worrier*; see Table 2), whereas GAD-7 comprises two items with the word *worrying* and one with *anxious*. This abbreviated version of the original PSWQ (Meyer et al., 1990; Hopko et al., 2003) is a frequently used measure of worry without the reversed coded items. The PSWQ-8 assesses pathological worry with comparable validity and reliability as the full 16-item version (Wuthrich et al., 2014). As for depression, the word-response question for worry captures individuals’ subjective experiences and their own understanding of the construct.

**TABLE 2:** DSM-5 diagnostic criteria for Generalized Anxiety Disorder.

**DSM-5 criteria**	**Primary**	**GAD-7 items**	**PSWQ-8 items**
Excessive anxiety and worry, for at least 6 months, about a number of events or activities.	Y	*Item 3*. Worrying too much about different things.	*Item 2.* Many situations make me worry. *Item 4.* When I am under pressure, I worry a lot.
Difficulties in controlling the worry.	Y	*Item 2*. Not being able to stop or control worrying.	*Item 1.* My worries overwhelm me *Item 3.* I know I should not worry about things, but I just cannot help it. *Item 5.* I am always worrying about something. *Item 6.* As soon as I finish one task, I start to worry about everything else I must do. *Item 7.* I have been a worrier all my life *Item 8.* I have been worrying about different things.
Three (or more) of the following six symptoms:			
(1) Restlessness, feeling keyed up or on edge.	N	*Item 1*. Feeling nervous, anxious, or on edge. *Item 5*. Being so restless that it’s hard to sit still	
(2) Being easily fatigued.	N	Not represented by any item.	
(3) Difficulty concentrating or mind going blank.	N	Not represented by any item.	
(4) Irritability.	N	*Item 6*. Becoming easily annoyed or irritable.	
(5) Muscle tension.	N	*Item 4*. Trouble relaxing.	
(6) Sleep disturbance.	N		
*- Felling afraid* is not included in the criteria for Generalized Anxiety Disorder 300.02 (F41.1)	N	*Item 7.* Feeling afraid as if something awful might happen.	

*Primary symptoms are marked with Y. GAD-7, General Anxiety Disorder-7 (Spitzer et al., 2006); PSWQ-8, Penn State Worry Questionnaire-8 (Hopko et al., 2003).*

### Aims and Hypotheses

This study extends research by Kjell et al. (2019) in two central ways. First, it aims to examine to what extent the QCLA method captures aspects related to the primary and secondary symptoms associated with MDD and GAD as captured by the items of the corresponding rating scale. To test this, we mapped the word responses to the individual items in the rating scales. The choice of rating scales was motivated because these scales are designed to target the DSM criteria for MDD and GAD (American Psychological Association [APA], 2013). Second, with two rating scales targeting anxiety, the GAD-7 (Spitzer et al., 2006) and the PSWQ-8 (Hopko et al., 2003), we further examined QCLA’s ability to capture primary symptoms associated with GAD. These aims are divided into the following three hypotheses.

#### The Semantic Hypothesis

To further understand the relationship between word responses and rating scales, we examined the correlations to individual items using semantic similarity scales (unipolar and bipolar), language-trained scales, and language-predicted valence scales. We hypothesize that the word embeddings of the word answers significantly capture all items of the depression and worry rating scales through semantic similarity scales and the language-trained scales.

#### The Valence Hypothesis

Given that the word embeddings for worry and depression words predict the items in the rating scales for the corresponding construct, we further investigated what specific information in the word embeddings contribute to the correlation. Kjell et al. (2019) argued that rating scales are highly influenced by valence, potentially capturing a more general negative feeling for depression and anxiety. Thus, we hypothesize that language-predicted valence scores are correlated with each individual item and that they can explain a substantial part of the correlation between language-trained scales and observed scales.

#### Primary Over Secondary Criteria Hypothesis

Because the word-response questions focus on respondents’ experiences and understanding of a construct, it is important to examine to what extent they also capture the secondary symptoms of the diagnostic criteria for MDD as measured by the PHQ-9 and the GAD as measured by the GAD-7. Based on the relatively general nature of the word-response question (i.e., it does not ask for related symptoms/behaviors of MDD/GAD), we hypothesize that the word embeddings from depression and worry word responses yield stronger correlations to items capturing the primary over the secondary criteria. For example, we anticipate that the semantic similarity scales of depression correlate stronger to the PHQ-9 item about feeling down or depressed (Item 2) than the item about psychomotor agitation or retardation (Item 8).

## Materials and Methods

### Participants

Mechanical Turk (MTurk^1^) was used to recruit participants. This platform enables participants to perform tasks, such as research studies, with an economic gain. MTurk has been used to study clinically relevant topics (Shapiro et al., 2013), with a prevalence of depression and anxiety corresponding to that of community samples [Shapiro et al., 2013; however, other studies suggest higher (Arditte et al., 2016) or lower levels (Veilleux et al., 2015)]. MTurk is generally more diverse than convenience samples such as student and community samples (Chandler and Shapiro, 2016). In our study, 455 respondents submitted their survey, and 44 (9.7%) were excluded from the analyses due to failure to answer the control items correctly (see section “Measures and Material” below). The final sample comprised 411 respondents (47% females, 53% males) ranging in age from 18 to 74 years (Mean = 36.2, *SD* = 11.2). Most participants were from the US (86%), followed by India (11%) and other countries (3%). Out of the 411 participants, 37% were above the cut-off point for MDD on the PHQ-9 (i.e., a score of 10 or higher), and 33% scored above the cut-off for GAD on the GAD-7 (i.e., a score of 10 or higher). These rates were higher than in the general population (Bromet et al., 2011). The participants’ reported average perceived personal financial situation was 4.57 (*SD* = 1.71) on a scale ranging from 1 = “Our income does not cover our needs, there are great difficulties” to 7 = “Our income covers our needs, we can save.” Participants were paid USD 1 to participate.

### Measures and Material

*The Word-Response Question of Depression* (Kjell et al., 2019) involves asking *Over the last 2 weeks, have you been depressed or not?* coupled with the instructions to answer with their own descriptive words. The instructions furthermore asked participants to “weigh the strength and the number of words” to describe their worry, to focus on writing important and meaningful aspects, and to only write one word in each of the five empty response boxes. Participants were asked to generate five descriptive words.

*The Word-Response Question of Worry* (Kjell et al., 2019) is coupled with an adapted version of the instructions for the word-response question of depression by changing *depression* to *worry*. i.e., *Over the last 2 weeks, have you been worried or not?* and required five descriptive words as the response format.

*The PHQ-9* (Kroenke et al., 2001) includes nine items such as *Feeling down, depressed, or hopeless* coupled with a closed-ended response format ranging from 0 = *Not at all* to 3 = *Nearly every day*. Participants are asked to consider the last 2 weeks. The PHQ-9 has been validated in primary care (Kroenke et al., 2001) and in the general population (Löwe et al., 2004; Martin et al., 2006; Stochl et al., 2020). Additionally, the PHQ-9 has demonstrated the ability to detect changes in response to treatment of various depressive disorders (Löwe et al., 2004). The scale demonstrated a McDonald’s ω of 0.94 and a Cronbach’s α of 0.92 in the current study.

*The GAD-7* (Spitzer et al., 2006) includes seven items such as *Worrying too much about different things* coupled with the same closed-ended response format and timeframe as the PHQ-9. The GAD-7 has been validated in primary care settings (Spitzer et al., 2006) and in the general population (Löwe et al., 2008). Additionally, the GAD-7 has shown sensitivity to detect changes in patients receiving treatment for GAD (Dear et al., 2011). The scale demonstrated a McDonald’s ω of 0.95 and a Cronbach’s α of 0.93 in the current study.

*The PSWQ-8* (Hopko et al., 2003) is an abbreviated version of the PSWQ (Meyer et al., 1990) and encompasses items such as *My worries overwhelm me*, with a closed-ended scale ranging from 0 = *Not at all typical of me* to 5 = *Very typical of me*. In contrast to the full 16-item version, the PSWQ-8 does not include any reverse-coded items. The PSWQ-8 has been validated in a sample of younger (Crittendon and Hopko, 2006) and older (Hopko et al., 2003) adults and a clinical sample of adults (Kertz et al., 2014). The scale yielded a McDonald’s ω of 0.97 and a Cronbach’s α of 0.96 in the current study.

*Control items* were randomly presented within the PHQ-9 and the GAD-7, including *On this question please answer the alternative ‘Several days,’ On this question please answer the alternative ‘More than half the days,’* and *On this question please answer the alternative ‘Not at all.’* Respondents who failed to answer *all* control items (in total two per participant) correctly were excluded from the analyses. Importantly, attention control items have been found to increase the statistical reliability of the data (e.g., see Oppenheimer et al., 2009; for the use of similar control items see Kjell et al., 2021a).

*The demographic survey* included questions regarding age, gender, country of origin, first language, and their perception of their household income. When asked about gender, participants were offered three alternatives: male, female, and other. Perceived financial situation was measured by asking, “Does the total income of your household allow you to cover your needs?” with the responses ranging from 1 = “Our income does not cover our needs, there are great difficulties,” to 7 = “Our income covers our needs, we can save.”

*The Word Norm for Depression* (Kjell et al., 2019) includes 1,172 words describing being depressed generated by asking 110 participants to describe their “view of being depressed” with 10 words. When constructing the word norm, the targeted word “depression” was also added, so it is the most frequently occurring word by 1 in the norm.

*The Word Norm for Worry* (Kjell et al., 2019) includes 1,036 words describing being depressed generated by asking 104 participants to describe their “view of being worried” with 10 words. When constructing the norm, the targeted word “worry” was added, so it is the most frequently occurring word by 1 in the norm.

*The Word Norm for Not at all Depressed* (Kjell et al., 2020) includes 1,125 words generated by 115 participants describing their “view of being not at all depressed.”

*The Word Norm for Not at all Worried* (Kjell et al., 2020) includes 938 words generated by 97 participants describing their “view of being not at all worried.”

*The language-predicted valence* scores were based on a model constructed from the ANEW (Bradley and Lang, 1999), which is a list of more than 1,000 words such as “cat” and “kindness” coupled with participant-rated valence scores ranging from unpleasant to pleasant. The model was created by training the word embeddings for the words in the ANEW list to their corresponding valence score, e.g., cat (*M* = 4.38, *SD* = 2.24). Using cross-validation leave-k-out (described below in more detail) produced a strong correlation between predicted and actual valence ratings (*r* = 0.74, *p* < 0.001, *N* = 1031 words). This computational model was applied to the word embeddings from the word-response questions in this study to estimate a language-predicted valence score.

### Procedure

Participants were informed that the study comprised questions regarding their mental health, including aspects such as depression and worry/anxiety and that they should answer with both descriptive words and rating scales. They were presented with the consent form that included details about how to receive more information, that their responses were recorded anonymously, and that they had the right to withdraw from the study at any time. The study started with the word-response questions presented in random order; followed by the corresponding rating scales in random order. The word-response questions were presented first to avoid the wordings of the rating-scale items from influencing the word responses. Lastly, the participants were asked to fill out the brief demographic survey. In the end, the participants were debriefed. The completion time was on average 10 min and 5 s. According to Swedish law, the National Ethics Committee (protocol number 2020-00730) reviewed the study and decided that it did not require ethical approval.

### Statistical Analyses

#### Natural Language Processing and Machine Learning

The QCLA approach encompasses various techniques to analyze word and text responses in relation to numeric rating scales (see Kjell et al., 2019). These techniques include natural language processing, machine learning, and statistics.

##### Word embeddings

To represent words with numbers, we used a semantic space from Semantic Excel^2^ (Sikström et al., 2018). This space, referred to as *English 1*, was created using an approach akin to latent semantic analysis, where a word co-occurrence table is generated, and the semantic space is produced by applying a data compression algorithm (i.e., SVD) on this table. Technical details on this can be found in Kjell et al. (2019), but see also Landauer and Dumais (1997). This space is generated from the English corpus Google 5-gram database consisting of 1.7 ^∗^ 10^9^ words (Version 20120701^3^). The generated space consists of the 120,000 most common words in the corpus where each word is represented by a vector consisting of 512 numbers describing how the words in the semantic space are semantically related to each other. This representation is referred to as a *word embedding*.

##### Responses

Participants’ responses were cleaned by changing the word spellings to American English using MS Word, and misspelled words were corrected in those cases where the meaning was clear. The word embeddings for the five words generated by each participant for a given word-response question were aggregated by taking the mean across the dimensions, so these words are represented by one word embedding that captures the meaning of the five words taken together in 512 dimensions.

##### Semantic similarity

A word embedding describes how a word, or set of words, in the semantic space is positioned in relation to all the other words. The closer two words are positioned, the more semantically similar they are. The semantic similarity between two words (or two sets of words) is computed as the cosine of the angle between the two word embeddings in the semantic space. The semantic similarity scores are mathematically bounded between –1 and +1, but in practice they tend to range between a value around 0 (for unrelated words) and a value significantly less than 1, where a higher value indicates higher semantic similarity.

##### Language training and prediction

The dimensions of the word embeddings may be used as predictors in a multiple regression to predict a numeric variable such as a rating scale. In multiple regression (i.e.,$y=\beta_{0}+\beta_{1}^{*}\,x_{1}\,\ldots \,\beta_{m}^{*}\,x_{m}+\varepsilon$), y is the observed variable (e.g., the rating scale score), *x* is the word embedding including several dimensions (i.e., *x*_1_,*x*_2_, etc.), β_*m*_ is the coefficient, β_*0*_ the intercept/constant, and ε the error term. For the machine learning implementation, we used the default settings in the *text*-package (version 0.9.10 from CRAN; Kjell et al., 2021b), which involves using ridge regression (Hoerl and Kennard, 1970) with a penalty search grid ranging from 10^–16^ to 10^16^ and a sequence of times 10. The penalty hyperparameter was tuned using 10-fold cross-validation, where the training set was further divided into an analysis (75%) and assessment (25%) set (see Kjell et al., 2021b). This cross-validation procedure enables a determination of the accuracy of the prediction. Here the predicted value (y∧) is correlated with the empirical value (y, i.e. the rating scale score), and the correlation coefficient (*r*) is the measure of accuracy.

##### Supervised dimension projection plot

The supervised dimension projection (SDP) plot from the *text*-package was used to visualize the word responses. The SDP plots words according to a dimension created by comparing two groups of words (e.g., depression versus worry responses or a quartile split on low versus high scorers on the PHQ-9). In short, the dimension is created by first aggregating all the word embeddings of all words in each group and then subtracting the two aggregated word embeddings to create the *aggregated direction embedding* that is seen to make up a line (dimension) running through origo. Subsequently, individual words’ embeddings are first positioned in relation to the mean word embedding of all words (i.e., their word embeddings are subtracted with this embedding) and then projected onto the dimension using the dot product. The *p*-value for each dot product score is computed using a permutation procedure (the default settings in *text* versions.9.10 were used; for more details see Kjell et al., 2021b).

#### Statistical Software and Packages

The analyses were carried out in R (R Core Team, 2020), where the word-related analyses were carried out using the *text* package (Kjell et al., 2020). Other analyses included using *tidyverse* (Wickham et al., 2019), *psych* (Revelle, 2017), and *Hmisc* (Harrell, 2017).

#### Interpreting Statistics: Statistical Cut-Off Points

To interpret the internal reliability of the rating scales as good, we used 0.70 as the cut-off for Cronbach’s α and McDonald’s ω. To interpret the correlation strengths, we used Cohen’s (1988) conventions of 0.10, 0.30, and 0.50 for a small/weak, moderate, and large/strong correlation, respectively. Alpha was set to 0.05. The sample size was based on the finding by Kjell et al. (2019) that between 256 and 477 yields correlations to aggregated rating scales that correlate above 0.50 (i.e., *r* > 0.5), which is sufficiently high for evaluating the hypotheses in this article (i.e., the aim here was not to maximize the accuracy but rather to understand the models).

## Results

### Descriptive Statistics

**FIGURE 1:**
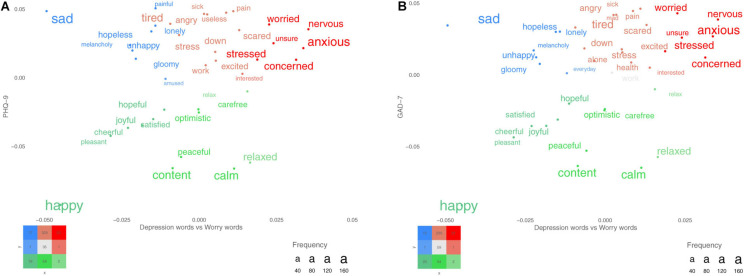
**(A,B)** Words that participants used for describing their depression and worry. The *x*-axes represent words differentiating between depression (left) and worry (right), and the *y*-axes represent the PHQ-9 total score for plot A and the GAD-7 total score for plot B. The colored words represent significant words when correcting for multiple comparisons using the false discovery rate (FDR). More frequent words are plotted with larger font size.

Descriptive statistics are presented in Table 3. The GAD-7 and the PHQ-9 yielded a positive skew and deviated from a normal distribution, whereas the PSWQ-8 demonstrated a normal distribution. Therefore, Spearman rho was applied for the GAD-7 and the PHQ-9 and Pearson’s *r* was applied for the PSWQ-8. The correlations among the total scores of the included measures are presented in Table 4. Figures 1A,B show participants’ word responses using SDP plots.

**TABLE 3:** Mean, standard deviation, range, skew, and kurtosis for rating and semantic scales.

**Measure**	***M***	***SD***	**Range**	**Skew**	**Kurtosis**
PHQ-9	8.41	6.96	27.00	0.51	–0.74
GAD-7	7.37	5.84	21.00	0.33	–0.97
PSWQ-8	25.01	9.87	32.00	–0.30	–1.07
SSS Worry bipolar	0.06	0.28	1.14	–0.67	–0.59
SSS Depression bipolar	–0.01	0.24	0.95	–0.24	–1.22
SSS Depression unipolar	0.28	0.16	0.71	0.51	–0.57
SSS Worry unipolar	0.33	0.19	0.83	0.33	–0.88
Language-trained PHQ-9	8.39	3.85	15.31	–0.49	–1.03
Language-trained GAD-7	7.36	3.04	15.13	–1.03	0.29
Language-trained PSWQ-8	25.03	5.91	24.86	–1.13	0.13
Predicted valence of dep. words	5.21	1.44	5.78	0.19	–1.16
Predicted valence of wor. words	5.04	1.28	5.82	0.26	–1.09

*PHQ-9, Patient Health Questionnaire-9; GAD-7, General Anxiety Disorder Scale-7; PSWQ-8, Penn State Worry Questionnaire Abbreviated; SSS, The Semantic Similarity Scale; Bipolar, the semantic similarity of the high norm minus the semantic similarity of the low norm.; Unipolar, the semantic similarity between semantic responses and a high (or low) word norm.; dep., depression; wor., worry.*

**TABLE 4:** Correlations among measures.

**Variable**	**1**	**2**	**3**	**4**	**5**	**6**	**7**	**8**	**9**
(1) PHQ-9*									
(2) GAD-7*	0.86***								
**(3) PSWQ-8**	0.69***	0.81***							
**(4) Dw: Bipolar**	0.60***	0.53***	0.51***						
**(5) Ww: Bipolar**	0.44***	0.50***	0.54***	0.55***					
**(6) Dw: Unipolar H**	0.24***	0.22***	0.23***	0.64***	0.40***				
**(7) Ww: Unipolar H**	0.23***	0.28***	0.31***	0.38***	0.81***	0.49***			
**(8) Dw: Valence**	−0.57***	−0.49***	−0.45***	−0.87***	−0.51***	−0.58***	−0.37***		
**(9) Ww: Valence**	−0.47***	−0.50***	−0.49***	−0.56***	−0.81***	−0.36***	−0.63***	0.52***	

***Indicates *p* < 0.001.

*For PHQ-9 and GAD-7 we used Spearman’s rho.

*PHQ-9, Patient Health Questionnaire-9; GAD-7, General Anxiety Disorder Scale-7; PSWQ-8, Penn State Worry Questionnaire Abbreviated; Dw, depression words; Ww, worry words; Bipolar, bipolar semantic similarity scale; Unipolar, unipolar semantic similarity scale; H, High; Valence, language-predicted scale ANEW valence.*

*Rows 1–7 of this correlation table are also presented in Kjell et al. (2020).*

### Item Level Analyses of Depression and the PHQ-9

#### The Semantic Hypothesis

All correlations between the depression bipolar scale and the individual items composing the PHQ-9 were significant and varied from moderate to strong in correlational strengths (Table 5; bipolar:ρ = 0.31–0.61). In comparison to the bipolar scale, the unipolar scale showed lower correlations to individual items (ρ = 0.11–0.31), with non-significant correlations for Item 8 (moving patterns) and Item 9 (self-harm).

**TABLE 5:** Spearman’s rho correlations between total and individual PHQ-9 items for the unipolar (high) and bipolar semantic similarity scales for depression.

**PHQ-9**	***M***	***SD***	**Item total correlation**	**Unipolar SSS depression**	**Bipolar SSS depression**
Total	8.41	6.69		0.24***	0.60***
Item 1 (little interest)	0.95	0.96	0.77***	0.20***	0.53***
Item 2 (feeling down)	1.01	0.99	0.80***	0.27***	0.61***
Item 3 (disrupted sleep)	1.16	1.02	0.67***	0.26***	0.49***
Item 4 (little energy)	1.26	0.99	0.72***	0.31***	0.55***
Item 5 (changed food habits)	0.91	1.00	0.74***	0.23***	0.50***
Item 6 (failure)	1.03	1.03	0.78***	0.16**	0.50***
Item 7 (no concentration)	0.93	1.02	0.77***	0.11*	0.44***
Item 8 (slow or restless)	0.58	0.87	0.62***	0.08	0.31***
Item 9 (self-harm)	0.58	0.93	0.68***	0.5	31***

N = 411.

*Indicates *p* < 0.05; **indicates *p* < 0.01; ***indicates *p* < 0.001.

*M and *SD* are the mean and standard deviation, respectively.*

*PHQ-9, Patient Health Questionaire-9; SSS, Semantic Similarity Scale.*

The results for language-trained PHQ scales were similar to the findings for semantic similarity scales. Training the responses for the word-response question of depression to the individual items composing the PHQ-9 yielded significant correlations ranging from weak to strong (ρ = 0.30–0.60; *p* < 0.001; see Table 6).

**TABLE 6:** The mean and standard deviation for the PHQ-9 and its individual items and their correlations to the language-trained scale and the language-predicted valence scale from the depression word responses.

**PHQ-9 items**	***M***	***SD***	**Language-trained PHQ**	**Language-predicted valence**	**Partial correlation controlling for valence**
PHQ-9 total	8.41	6.96	0.60***	−0.57***	0.29***
Item 1 (little interest)	0.95	0.96	0.54***	−0.50***	0.29***
Item 2 (feeling down)	1.01	0.99	0.60***	−0.57***	0.28***
Item 3 (disturbed sleep)	1.16	1.02	0.44***	−0.46***	0.12*
Item 4 (little energy)	1.26	0.99	0.51***	−0.50***	0.21***
Item 5 (changed food habits)	0.91	1.00	0.47***	−0.44***	0.23***
Item 6 (failure)	1.03	1.03	0.50***	−0.49***	0.23***
Item 7 (no concentration)	0.93	1.02	0.45***	−0.40***	0.26***
Item 8 (slow or restless)	0.58	0.87	0.30***	−0.30***	0.15**
Item 9 (self-harm)	0.58	0.93	0.38***	−0.34***	0.22***

N = 411.

*Indicates *p* < 0.05; **indicates *p* < 0.01; ***indicates *p* < 0.001.

*Spearman’s rho, using Holms correction for multiple comparison. *M* and *SD* are the mean and standard deviation, respectively; PHQ-9, Patient Health Questionnaire-9, with item numbers corresponding to the order in Kroenke et al. (2001).*

*Partial correlation controlling for valence = Partialling out the language-predicted valance between the correlation of the language-trained PHQ score and the observed PHQ score.*

#### The Valence Hypothesis

The word responses’ language-predicted valence scale also correlated significantly with each PHQ-9 item (ρ = –0.30 to –0.57, *p* < 0.001). Absolute values for individual items except for Items 3 and 8 were stronger for the language-trained scales compared with the language-predicted valence scale. Controlling for valence reduced the language-trained item level correlations substantially from mean ρ = 0.47 (range:ρ = 0.30–0.60) to mean ρ = 0.22 (range: ρ = 0.12–0.29, see Table 6).

#### The Primary Over Secondary Criteria Hypothesis

The highest correlation for both the unipolar and the bipolar scale was to the item tapping into feeling down and depressed (Item 2). The lowest correlation for the unipolar scale was to the item targeting concentration difficulties (Item 7). The lowest corelations for the bipolar scale were to moving patterns (Item 8) and self-harm (Item 9). Changes in moving pattern (Item 8) and thoughts about being better off dead and self-harm (Item 9) were significant for the bipolar semantic similarity scale but not for the unipolar semantic similarity scale. The strongest correlations for both language-trained scales and language-predicted valence scales were to an item tapping into the emotional experience of depression (i.e., feeling down; ρ = 0.60 and –0.57, respectively). The lower correlations for both language-trained scales and language-predicted valence scales tended to be to behaviors relating to depression; for example, see Item 8 (moving pattern;ρ = 0.30 and –0.30, respectively) and Item 9 (self-harm;ρ = 0.38 and –0.34, respectively).

### Item Level Analyses of Worry and the GAD-7 and the PSWQ-8

#### The Semantic Hypothesis

All correlations of the worry unipolar and bipolar scales to each of the individual items composing the GAD-7 were significant and ranged from weak to strong (Table 7; unipolar:ρ = 0.16–0.32; bipolar:ρ = 0.32–0.50). All items composing the PSWQ-8 also correlated significantly with the worry unipolar and bipolar scales (Table 8; unipolar *r* = 0.26–0.34; bipolar: *r* = 0.52–0.61).

**TABLE 7:** Spearman’s rho correlations between total and individual GAD-7 items and the unipolar (high) and bipolar semantic similarity scales for worry.

**GAD-7**	***M***	***SD***	**Item total correlation for GAD-7**	**Unipolar SSS Worry**	**Bipolar SSS Worry**
Total	7.37	5.84	−	0.28***	0.50***
Item 1 (anxious, on edge)	1.13	0.97	0.88***	0.32***	0.50***
Item 2 (cannot control worrying)	1.09	1.03	0.90***	0.26***	0.47***
Item 3 (worrying about different things)	1.15	1.00	0.90***	0.27***	0.47***
Item 4 (trouble relaxing)	1.14	1.00	0.86***	0.21***	0.41***
Item 5 (restlessness, hard to sit still)	0.84	0.96	0.79***	0.16**	0.32***
Item 6 (annoyed, irritable)	1.05	0.94	0.78***	0.24***	0.42***
Item 7 (afraid)	0.97	0.99	0.85***	0.21***	0.41***

N = 411.

**Indicates *p* < 0.01; ***indicates *p* < 0.001.

*M and *SD* are the mean and standard deviation, respectively.*

*GAD-7, Generalised Anxiety Disorder Scale-7; SSS, Semantic Similarity Scale; item numbers correspond to the order in Spitzer et al. (2006).*

**TABLE 8:** Pearson correlations between total and individual PSWQ-8 items the unipolar (high) and bipolar semantic similarity scales for worry.

**PSWQ-8 items**	***M***	***SD***	**Item total correlation**	**Unipolar SSS Worry**	**Bipolar SSS Worry**
Total	25.01	9.87		0.33***	0.6***
Item 1 (worries overwhelming)	2.96	1.39	0.87***	0.27***	0.58***
Item 2 (situations worry)	3.15	1.38	0.91***	0.28***	0.55***
Item 3 (worry different things)	3.24	1.41	0.89***	0.30***	0.60***
Item 4 (pressure, worry)	3.39	1.32	0.86***	0.31***	0.55***
Item 5 (always worrying)	3.03	1.43	0.93***	0.30***	0.57***
Item 6 (after task, worry about new)	2.96	1.36	0.85***	0.28***	0.54***
Item 7 (worrier all my life)	3.08	1.47	0.85***	0.26***	0.52***
Item 8 (worrying about things)	3.21	1.38	0.87***	0.33***	0.53***

N = 411.

***Indicates *p* < 0.001.

*M and *SD* are the mean and standard deviation, respectively.*

*PSWQ-8, Penn State Worry Questionnaire Abbreviated; SSS, Semantic Similarity Scale; item numbers correspond to the order in Crittendon and Hopko (2006).*

The results from training the worry word responses to the individual items of the GAD-7 showed overall moderate correlations ranging from ρ = 0.41 to **0.52** (*p* < 0.001; see Table 9), and training to the items composing the PSWQ-8 resulted in moderate correlations ranging from *r* = 0.52 to **0.63** (*p* < 0.001; see Table 10).

**TABLE 9:** The mean and standard deviation for the GAD-7 and its individual items and their correlations to the language-trained scale and the language-predicted valence scale from the worry word responses.

**GAD-7 items**	***M***	***SD***	**Language-trained GAD**	**Language-predicted valence**	**Partial correlation controlling for valence**
GAD-7 total	7.37	5.84	0.54***	−0.50***	0.29***
Item 1 (anxious, on edge)	1.13	0.97	0.49***	−0.47***	0.24***
Item 2 (excessive worrying)	1.09	1.03	0.52***	−0.49***	0.25***
Item 3 (different areas of worry)	1.15	1.00	0.50***	−0.50***	0.19***
Item 4 (trouble relaxing)	1.14	1.00	0.41***	−0.39***	0.21***
Item 5 (restlessness)	0.84	0.96	0.42***	−0.31***	0.31***
Item 6 (annoyed, irritable)	1.05	0.94	0.42***	−0.40***	0.20***
Item 7 (afraid)	0.97	0.99	0.45***	−0.44***	0.23***

N = 411.

***Indicates *p* < 0.001.

*Spearman’s rho, using Holms correction for multiple comparison. *M* and *SD* are the mean and standard deviation, respectively.*

*Ww, worry words, GAD-7, Generalised Anxiety Disorder Scale-7, item numbers correspond to the order in Spitzer et al. (2006). Partial correlation controlling for valence = Partialling out the language-predicted valance between the correlation of language-trained GAD-7 score and observed GAD score.*

**TABLE 10:** The mean and standard deviation for the PSWQ-8 and its individual items and their correlations to the language-trained scale and the language-predicted valence scale from the worry word responses.

**PSWQ-8 items**	***M***	***SD***	**Language-trained PSWQ-8**	**Language-predicted valence**	**Partial correlation controlling for valence**
PSWQ-8 total	25.01	0.87	0.66***	−0.54***	0.46***
Item 1 (worries are overwhelming)	0.97	0.97	0.59***	−0.49***	0.39***
Item 2 (worrying about situations)	3.15	1.38	0.56***	−0.45***	0.39***
Item 3 (worrying about different things)	3.24	1.41	0.61***	−0.51***	0.40***
Item 4 (pressure, worry)	3.39	1.32	0.56***	−0.46***	0.37***
Item 5 (always worrying)	3.03	1.43	0.59***	−0.49***	0.39***
Item 6 (after completing a task, worrying about the next one)	2.96	1.36	0.56***	−0.45***	0.38***
Item 7 (worrier all my life)	3.08	1.47	0.52***	−0.42***	0.34***
Item 8 (worrying about things)	3.21	1.38	0.63***	−0.55***	0.39***

N = 411.

***Indicates p < 0.001.

*Pearson correlation, using Holms correction for multiple comparison. M and SD are the mean and standard deviation, respectively.*

*Ww, worry words; PSWQ-8, Penn State Worry Questionnaire Abbreviated, with item numbers corresponding to the order in Crittendon and Hopko (2006); Partial correlation controlling for valence = Partialling out the language predicted valance between the correlation of the language-trained PSWQ score and the observed PSWQ score.*

#### The Valence Hypothesis

The language-predicted valence scale from the worry responses also showed significant correlations to each of the individual items of the GAD-7 (*r* = –0.31 to –0.50) and the PSWQ-8 items (*r* = –0.42 to *r* = –0.55, *p* < 0.001). Controlling for valence using partial correlation reduced the language-trained item level correlations substantially for GAD-7 [mean ρ = 0.46 (range ρ = 0.41–0.52) to meanρ = 0.23 (range ρ = 0.19 –0.31)] and for PSWQ-8 [mean *r* = 0.58 (range *r* = 0.52–0.59) to mean *r* = 0.38 (range *r* = 0.37–0.40)].

### Primary Over Secondary Criteria Hypothesis

The highest correlations for both the unipolar and the bipolar scales were to the GAD-7 item representing the primary criterion about *feeling anxious and on edge* (Item 1) followed by the items about *worrying too much about different things* (Item 3) and worry *not being able to stop or control worrying* (Item 2). The lowest correlation was to the GAD-7 item tapping into the secondary criterion regarding *difficulties sitting still* (Item 5; i.e., a behavior).

For the PSWQ-8, all eight items demonstrated similar strengths (which were comparable in magnitude to the strongest items in the GAD-7). This consistency in strength and the comparably strong correlation make sense considering that all items are quite similar, tapping into the primary criterion of excessive worrying with different forms of the word “worry.” The highest correlation for both the unipolar and bipolar worry scale was in relation to Item 8, which targets the general tendency to worry.

In terms of the language-trained GAD-7 and language-predicted valence scales, the strongest correlations were to the item tapping into experiencing worry in different areas in life (ρ = 0.50 and –0.50 for the language-trained scales and the language-predicted valence scale, respectively). The lowest correlation was to items representing the secondary criteria about trouble relaxing (Item 4, ρ = 0.41 and –0.39) and being so restless that one finds it difficult to sit still (Item 5, ρ = 0.42 and –0.31), which can be seen as a related behavior to the experience of worry.

## Discussion

### The Semantic Hypothesis

We examined the relationship between word responses and rating scales by correlating the individual items of the respective rating scales with language-trained scales and semantic similarity scales (unipolar and bipolar). The semantic hypothesis was supported for the language-trained scales and the bipolar semantic similarity scales for the PHQ-9, the GAD-7, and the PSWQ-8. The unipolar semantic similarity scales correlated significantly for the GAD-7 and the PSWQ-8 items, but not for all items of the PHQ-9. Overall, these findings suggest that word embeddings capture the diagnostics criteria for MDD and GAD as measured by two of three QCLAs.

### The Valence Hypothesis

In accordance with the valence hypothesis, the language-predicted valence scales correlated significantly with each of the items composing the PHQ-9, the GAD-7, and the PSWQ-8. The language predicted valence scale overall tended to show comparable correlations to all items comprising the PHQ-9, equal or lower correlations to the GAD-7 and the PSWQ-8 items as compared with the language trained scales. A large part of the language-trained correlation was accounted for by the language-predicted valence scales; however, a significant portion of the correlation remained for all items. The results suggest that valence is a potentially strong contributor carrying a large part of the information for predicting rating scale items.

### The Primary Over Secondary Criteria Hypothesis

The hypothesis that the QCLA word-response would capture the primary criteria – i.e., the subjective experiences (such as thoughts and feelings) – better than secondary criteria (often more behavioral aspects) was supported. For the depression semantic similarity scales, the strongest correlation was to an item about feeling tired and having little energy. For the language-trained scales for depression, the strongest correlation was to the item tapping into feeling depressed, down, and hopeless, whereas the weakest correlations for both the language-trained scale and the semantic similarity scale for depression were to the items about related behavioral symptoms, including having trouble concentrating (Item 7), moving slowly or being fidgety (Item 8), and thoughts about being better off dead or self-harming (Item 9).

Further, it appears that the semantic similarity scales for worry primarily corresponded to the aspects of the rating scales that capture the primary criteria and the subjective experiences and understandings of worry, and less so the secondary criteria (especially the related behaviors). That is, the unipolar and bipolar scales correlated strongest to the GAD-7 items about feeling anxious and worry (Items 1–3) and less strongly to items tapping into related behaviors such as not being able to sit still (Item 5). The semantic similarity scale for worry captures all the items in the PSWQ-8 to a similar degree, which could be explained by all of the items directly asking about *worry*, *worrying*, *worries*, or being a *worrier*.

Overall, these results support the primary over secondary criteria hypothesis that the QCLA correlates stronger with items tapping into the cognitive experience of worry rather than with related behaviors. Thus, the QCLA appears to primarily capture the subjective experience of MDD and GAD, whereas when related behaviors appear to be captured they are covered less well. This is noteworthy from a clinical perspective and suggests that future studies are warranted to test specific word-response questions or word norms to capture behavioral aspects of the DSM-5 criteria for MDD and GAD. These findings suggest that future research should also consider developing word-response questions and/or word norms that more accurately capture secondary symptoms. For example, to measure the related behavior of self-harm, one could develop a word-response question that explicitly asks individuals to describe whether they self-harm or not, or alternatively creating a word norm comprising words related to self-harm and applying it to the word responses of the question on depression.

### Potential for Clinical Significance

The QCLA method allows participants to directly express and describe their experiences freely. In addition, the QCLA method does not prime patients with symptoms that the patient does not necessarily have or that are irrelevant for the patient. Thus, QCLA may have the potential to add value to clinical research and practices by complementing traditional rating scales and enhancing our understanding of patients’ experiences.

Research suggests that rating scales of depression tend to fail in reliably capturing the disorder across scales and that the scales miss important symptoms. For example, a literature review points out that seven commonly used rating scales for depression do not necessarily measure the same disorder because these scales include items that are aimed to measure a wide range of different symptoms (Van Loo et al., 2012; Fried, 2017). From this it follows that constructs do not generalize across scales that are aimed to measure the same disorder. Furthermore, to identify symptoms related to depression that matter for patients, Chevance et al. (2020) asked participants to describe “the most difficult aspect of depression to live with or endure?”. They found that the most frequently mentioned symptom was “mental pain,” which is missing in the DSM-5 criteria and in the closed-ended rating scale for depression used in this study. Importantly, the QCLA method offers an opportunity to examine the presence of symptoms beyond primary and secondary criteria (e.g., note that *painful* is one of the descriptive words related to high depression and depression responses in Figure 1A and that *pain* is related to both high PHQ-9 and GAD-7 in Figures 1A,B).

#### Semantic Similarity and Valence

In clinical settings, the language-predicted valence scales may potentially complement the semantic similarity scales in important ways. Language-predicted valence scores could be used to signal that further investigations are required even though the semantic similarity score is low (i.e., outside established cut-off points). That is, when answering a word-response question a person may use words that are comparably distant from the targeted word norm, but that from a mental health perspective warrant further investigation. In the cases where words have a negative valence, the language-predicted valence scale may be used to alert further attention. For example, to the word-response question for worry a patient may answer something that is semantically relatively far away from the worry word norm because it is related to, for example, depression; however, alarming words will often have a negative valence. Hence, if word responses have a low semantic similarity but a high negative valence score, this signals that the respondent’s answer should be investigated more closely and that another set of word-response questions may be needed.

#### Language Models and Objective Measures

We argue that language-trained scales are valuable when investigating the relationship between word-responses and numerical rating scales. In clinical settings, language-trained scales and predictions will potentially be very important given that they can be trained and used to predict objective measures and outcomes rather than self-reported numerical rating scales. That is, word responses may be trained to actual behavior such as sick leaves, number of suicide attempts, or information obtained from smartphone apps including quality of sleep, walking speed, etc. [e.g., see Miller (2012) for ways to collect data with smartphones]. These language-trained models can potentially be used to investigate the relationship between word responses and the objective measure as well as to predict future respondents’ behaviors with the possibility to tailor treatment interventions.

### Limitations

It is important to note that this study used *individual* items to examine the degree that QCLAs capture symptoms; hence, future research could use specific assessments (rather than just one item) and/or objective measures (e.g., of sleep) to examine this further. Future studies could also examine potential benefits from using recent language models that can take word order (i.e., context) into account (e.g., Devlin et al., 2019), which is an improvement by analyzing descriptive texts as compared to descriptive words (Kjell et al., in progress).

MTurk is an efficient way to collect data from individuals with a wide range of backgrounds. It should be noted that generalization from MTurk should be made carefully; however, previous MTurk studies have been shown to be more representative compared with other samples commonly used in clinical research (e.g., Chandler and Shapiro, 2016). Lastly, this study did not collect data that allows for analysis of attrition (i.e., only data where participants completed the entire survey was collected), which further emphasizes the importance of being careful when generalizing the results.

## Conclusion

The QCLAs (i.e., unipolar, bipolar, language trained, and language-predicted valence scales) cover all aspects of the rating scale items that are designed to cover the primary DSM criteria as measured by rating scales, although with strengths varying from weak to strong. The QCLAs appear specifically suited to capture an individual’s cognitive and emotional experiences of depression, worry, and anxiety. Overall, they also capture the secondary criteria (generally including more behaviors and physiological symptoms) related to these experiences as measured by the rating scales. We believe that the QCLAs could be of great importance for clinical research and practice, where word-responses are coupled with objectively measured outcomes. Further, because the QCLA method is based on the respondent’s own descriptions of their experiences and symptoms related to a construct, the method carries the potential to personalize assessments, which might contribute to the ongoing discussion regarding the diagnostic heterogeneity of depression.

## Data Availability Statement

Data will not be made freely available as participants where not informed about this, and these open-ended text responses may be a risk for identification.

## Ethics Statement

The study was reviewed by the Ethics Review Authority who decided that it did not require ethical approval. The participants provided their written informed consent to participate in this study.

## Author Contributions

KK has stated the hypotheses, collected the data, performed the analysis, and written the manuscript with the support and guidance from SS. PJ contributed to help the stating hypothesis and gave feedback to the manuscript. All authors contributed to the article and approved the submitted version.

## Conflict of Interest

KK and SS co-founded WordDiagnostics focusing on diagnosing psychiatric disorders using language based assessments. The remaining author declares that the research was conducted in the absence of any commercial or financial relationships that could be construed as a potential conflict of interest.

## Footnotes

1 www.mturk.com

2 www.SemanticExcel.com

3 https://books.google.com/ngrams

## References

[B1] American Psychological Association [APA] (2013). *Diagnostic and Statistical Manual of Mental Disorders (DSM-5).* Washington, DC: American Psychiatric Association.

[B2] Arditte K. A. Çek D. Shaw A. M. Timpano K. R. (2016). The importance of assessing clinical phenomena in Mechanical Turk research. *Psychol. Assessm.* 28:684. 10.1037/pas0000217 26302105

[B3] Bradley M. M. Lang P. J. (1999). *Affective Norms for English Words (ANEW): INSTRUCTION MANUAL and Affective Ratings.* Technical Report C-1. Florida: The Center for Research in Psychophysiology, University of Florida.

[B4] Bromet E. Andrade L. H. Hwang I. Sampson N. A. Alonso J. De Girolamo G. (2011). Cross-national epidemiology of DSM-IV major depressive episode. *BMC Med.* 9:90. 10.1186/1741-7015-9-90 21791035 PMC3163615

[B5] Chandler J. Shapiro D. (2016). Conducting clinical research using crowdsourced convenience samples. *Annu. Rev. Clin. Psychol.* 12 53–81. 10.1146/annurev-clinpsy-021815-093623 26772208

[B6] Chevance A. Ravaud P. Tomlinson A. Le Berre C. Teufer B. Touboul S. (2020). Identifying outcomes for depression that matter to patients, informal caregivers, and health-care professionals: qualitative content analysis of a large international online survey. *Lancet Psychiatry* 7 692–702. 32711710 10.1016/S2215-0366(20)30191-7

[B7] Cohen J. (1988). *Statistical Power Analysis for the Behavioralbehavioural Sciences.* Hilsdale, NJ: Lawrence Earlbaum Associates.

[B8] Crittendon J. Hopko D. R. (2006). Assessing worry in older and younger adults: psychometric properties of an abbreviated Penn State Worry Questionnaire (PSWQ-A). *J. Anxiety Disord.* 20 1036–1054. 16387472 10.1016/j.janxdis.2005.11.006

[B9] de Beurs E. den Hollander-Gijsman M. E. van Rood Y. R. van der Wee N. J. Giltay E. J. van Noorden M. S. (2011). Routine outcome monitoring in the Netherlands: practical experiences with a web- based strategy for the assessment of treatment outcome in clinical practice. *Clin. Psychol. Psychother.* 18 1–12. 20238371 10.1002/cpp.696

[B10] De Choudhury M. Gamon M. Counts S. Horvitz E. (2013). “Predicting depression via social media,” in *Proceedings of the Seventh International AAAI Conference on Weblogs and Social Media ICWSM-13*, New York, NY.

[B11] Dear B. F. Titov N. Sunderland M. McMillan D. Anderson T. Lorian C. (2011). Psychometric comparison of the generalized anxiety disorder scale-7 and the Penn State Worry Questionnaire for measuring response during treatment of generalised anxiety disorder. *Cogn. Behav. Therapy* 40 216–227. 10.1080/16506073.2011.582138 21770844

[B12] Devlin J. Chang M. W. Lee K. Toutanova K. (2019). Bert: pre-training of deep bidirectional transformers for language understanding. *arXiv* [Preprint]. Available online at: https://arxiv.org/abs/1810.04805

[B13] Eichstaedt J. C. Smith R. J. Merchant R. M. Ungar L. H. Crutchley P. Preoţiuc-Pietro D. (2018). Facebook language predicts depression in medical records. *Proc. Natl. Acad. Sci. U.S.A.* 115 11203–11208. 30322910 10.1073/pnas.1802331115 PMC6217418

[B14] Fried E. I. (2017). The 52 symptoms of major depression: lack of content overlap among seven common depression scales. *J. Affect. Disord.* 208 191–197. 27792962 10.1016/j.jad.2016.10.019

[B15] Harrell F. E. Jr. (2017). *Hmisc: Harrell Miscellaneous. R Package Version 4.0-3.* Available online at: https://CRAN.R-project.org/package=Hmisc

[B16] Hoerl A. E. Kennard R. W. (1970). Ridge regression: biased estimation for nonorthogonal problems. *Technometrics* 12 55–67.

[B17] Hopko D. R. Reas D. L. Beck J. G. Stanley M. A. Wetherell J. L. Novy D. M. (2003). Assessing worry in older adults: confirmatory factor analysis of the Penn state worry questionnaire and psychometric properties of an abbreviated model. *Psychol. Assess.* 15:173. 12847777 10.1037/1040-3590.15.2.173

[B18] Kertz S. J. Lee J. Björgvinsson T. (2014). Psychometric properties of abbreviated and ultra-brief versions of the Penn State Worry Questionnaire. *Psychol. Assess.* 26:1146. 24932640 10.1037/a0037251

[B19] Kesebir P. Diener E. (2008). In pursuit of happiness empirical answers to philosophical questions. *Perspect. Psychol. Sci.* 3 117–125. 10.1111/j.1745-6916.2008.00069.x 26158878

[B20] Kjell O. N. Diener E. (2020). Abbreviated three-item versions of the satisfaction with life scale and the harmony in life scale yield as strong psychometric properties as the original scales. *J. Pers. Assess.* 103 183–194. 32167788 10.1080/00223891.2020.1737093

[B21] Kjell O. N. E. Daukantaitë D. Sikstroöm S. (2021a). Computational language assessments of harmony in life -not satisfaction with life nor rating scales - correlate with cooperative behaviors. *Front. Spec. Issue Seman. Algor. Assess. Attitud. Personal.* 10.3389/fpsyg.2021.601679 34045988 PMC8144476

[B22] Kjell O. N. E. Kjell K. Garcia D. Sikström S. (2019). Semantic measures: using natural language processing to measure, differentiate, and describe psychological constructs. *Psychol. Methods* 24 92–115. 29963879 10.1037/met0000191

[B23] Kjell O. N. E. Kjell K. Garcia D. Sikström S. (2020). “Semantic similarity scales: using semantic similarity scales to measure depression and worry,” in *Statistical Semantics*, eds Sikström S. Garcia D. (Cham: Springer), 10.1007/978-3-030-37250-7_4

[B24] Kjell O. N. E. Giorgi S. Schwartz H. A. (2021b). *Text: An R-Package for Analyzing and Visualizing Human Language Using Natural Language Processing and Deep Learning*. Available online at: 10.31234/osf.io/293kt (accessed April 16, 2021). PMC12803442 37126041

[B25] Kjell O. N. E. Sikström S. Kjell K. Schwartz A. (in progress) *Question Based Text Responses Analysed with AI-Based Transformer Approach the Upper Limits of Rating Scale Reliability.*

[B26] Kroenke K. Spitzer R. L. Williams J. W. B. (2001). The PHQ-9: validity of a brief depression severity measure [Electronic version]. *J. Gen. Intern. Med.* 16 606–613. 11556941 10.1046/j.1525-1497.2001.016009606.x PMC1495268

[B27] Landauer T. K. Dumais S. T. (1997). A solution to Plato’s problem: the latent semantic analysis theory of acquisition, induction, and representation of knowledge. *Psychol. Rev.* 104 211–240. 10.1037/0033-295x.104.2.211

[B28] Löwe B. Decker O. Müller S. Brähler E. Schellberg D. Herzog W. (2008). Validation and standardization of the Generalized Anxiety Disorder Screener (GAD-7) in the general population. *Med. Care* 46 266–274. 18388841 10.1097/MLR.0b013e318160d093

[B29] Löwe B. Unützer J. Callahan C. M. Perkins A. J. Kroenke K. (2004). Monitoring depression treatment outcomes with the patient health questionnaire-9. *Med. Care* 42 1194–1201. 15550799 10.1097/00005650-200412000-00006

[B30] Martin A. Rief W. Klaiberg A. Braehler E. (2006). Validity of the brief patient health questionnaire mood scale (PHQ-9) in the general population. *Gen. Hosp. Psychiatry* 28 71–77. 16377369 10.1016/j.genhosppsych.2005.07.003

[B31] Meyer T. J. Miller M. L. Metzger R. L. Borkovec T. D. (1990). Development and validation of the penn state worry questionnaire. *Behav. Res. Therapy* 28 487–495. 10.1016/0005-7967(90)90135-6 2076086

[B32] Miller G. (2012). The smartphone psychology manifesto. *Perspect. Psychol. Sci.* 7 221–237. 26168460 10.1177/1745691612441215

[B33] Mowery D. Smith H. A. Cheney T. Bryan C. Conway M. (2016). Identifying depression-related tweets from twitter for public health monitoring. *Online J. Public Health Inform.* 8:6561. 10.5210/ojphi.v8i1.6561

[B34] Oppenheimer D. M. Meyvis T. Davidenko N. (2009). Instructional manipulation checks: detecting satisficing to increase statistical power. *J. Exp. Soc. Psychol.* 45 867–872.

[B35] R Core Team (2020). *R: A Language and Environment for Statistical Computing.* Vienna: R Foundation for Statistical Computing.

[B36] Reece A. G. Reagan A. J. Lix K. L. M. Dodds P. S. Danforth C. M. Langer E. J. (2017). Forecasting the onset and course of mental illness with Twitter data. *Sci. Rep.* 7:13006. 10.1038/s41598-017-12961-9 29021528 PMC5636873

[B37] Revelle W. (2017). *Psych: Procedures for Personality and Psychological Research.* Evanston, IL: Northwestern University.

[B38] Shapiro D. N. Chandler J. Mueller P. A. (2013). Using mechanical turk to study clinical populations. *Clin. Psychol. Sci.* 1 213–220.

[B39] Sikström S. Kjell O. N. E. Kjell K. (2018). *Semantic Excel: An Introduction to a User-Friendly Online Software Application for Statistical Analyses of Text Data*. Available online at: 10.31234/osf.io/z9chp (accessed October 25, 2018).

[B40] Spitzer R. L. Kroenke K. Williams J. W. B. Löwe B. (2006). A brief measure for assessing generalized anxiety disorder: the gad-7. *Archiv. Intern. Med.* 166 1092–1097. 10.1001/archinte.166.10.1092 16717171

[B41] Stochl J. Fried E. I Fritz J. Croudace T. J. Russo D. A. Knight C. (2020). On dimensionality, measurement invariance, and suitability of sum scores for the PHQ-9 and the GAD-7. *Assessment* 10.1177/1073191120976863 33269612

[B42] Van Loo H. M. De Jonge P. Romeijn J. W. Kessler R. C. Schoevers R. A. (2012). Data-driven subtypes of major depressive disorder: a systematic review. *BMC Med.* 10:156. 10.1186/1741-7015-10-156 23210727 PMC3566979

[B43] Veilleux J. C. Salomaa A. C. Shaver J. A. Zielinski M. J. Pollert G. A. (2015). Multidimensional assessment of beliefs about emotion: development and validation of the emotion and regulation beliefs scale. *Assessment* 22 86–100. 24835246 10.1177/1073191114534883

[B44] Washington A. E. Lipstein S. H. (2011). The patient-centered outcomes research institute–promoting better information, decisions, and health. *N. Engl. J. Med.* 365:e31. 21992473 10.1056/NEJMp1109407

[B45] Wickham H. Averick M. Bryan J. Chang W. D’Agostino L. McGowan L. (2019). Welcome to the tidyverse. *J. Open Source Softw.* 4:1686.

[B46] Wuthrich V. M. Johnco C. Knight A. (2014). Comparison of the Penn State Worry Questionnaire (PSWQ) and abbreviated version (PSWQ-A) in a clinical and non-clinical population of older adults. *J. Anx. Disord.* 28 657–663. 10.1016/j.janxdis.2014.07.005 25124502

